# Customisation of the Exome Data Analysis Pipeline Using a Combinatorial Approach

**DOI:** 10.1371/journal.pone.0030080

**Published:** 2012-01-06

**Authors:** Swetansu Pattnaik, Srividya Vaidyanathan, Durgad G. Pooja, Sa Deepak, Binay Panda

**Affiliations:** 1 Ganit Labs, Bio-IT Centre, Institute of Bioinformatics and Applied Biotechnology, Bangalore, India; 2 Strand Life Sciences, Bangalore, India; University of Turin, Italy

## Abstract

The advent of next generation sequencing (NGS) technologies have revolutionised the way biologists produce, analyse and interpret data. Although NGS platforms provide a cost-effective way to discover genome-wide variants from a single experiment, variants discovered by NGS need follow up validation due to the high error rates associated with various sequencing chemistries. Recently, whole exome sequencing has been proposed as an affordable option compared to whole genome runs but it still requires follow up validation of all the novel exomic variants. Customarily, a consensus approach is used to overcome the systematic errors inherent to the sequencing technology, alignment and post alignment variant detection algorithms. However, the aforementioned approach warrants the use of multiple sequencing chemistry, multiple alignment tools, multiple variant callers which may not be viable in terms of time and money for individual investigators with limited informatics know-how. Biologists often lack the requisite training to deal with the huge amount of data produced by NGS runs and face difficulty in choosing from the list of freely available analytical tools for NGS data analysis. Hence, there is a need to customise the NGS data analysis pipeline to preferentially retain true variants by minimising the incidence of false positives and make the choice of right analytical tools easier. To this end, we have sampled different freely available tools used at the alignment and post alignment stage suggesting the use of the most suitable combination determined by a simple framework of pre-existing metrics to create significant datasets.

## Introduction

DNA Sequencing has come a long way since its first discovered more than 30 years back, in terms of speed, throughput and cost. The commercial availability of second generation sequencing technology has aided many new discoveries, especially in the field of disease biology, microbiology and plant biology. As amount of data generated from the next generation sequencing (NGS) platforms is very large that requires sophisticated informatics tools and skills in computational biology to mine, analyse and interpret the data, bulk of the research in the field has come from a handful of large genome centres that employ large numbers of computer scientists, computational biologists and bioinformatics specialists. A small biology lab with limited resources in informatics, software and hardware usually find it difficult to analyse NGS data. Additionally, variants discovered with NGS platforms often need downstream biological validation. In order to get the best set of variants, one needs to use the right combination of tools to discover them in the first place, reducing false positive calls due to amplification bias and sequencing error. In order to make NGS technology ubiquitous and clinically useful, one needs to come up with simplified analysis tools that produce more true positive calls and reduces efforts and money required for downstream validation experiments. The trajectory between NGS data generation and biological meaning currently spans multiple known and approximate landscapes with dimensionality defined grossly by factors like data-compression, string matching, and consensus building among others. The approximation seeds mainly from the universal assumption ingrained in the alignment algorithms that the number of expected mismatches be governed by the genetic polymorphism rate of the species/population and the systematic error rate in the sequencing technology rather than by considerations of evolutionary substitutions/mutations [1]. Also, calculation of alignment maps without increase in computer hardware requirements for high throughput data has been the central theme for optimization of most alignment algorithms necessitating the use of approximate heuristic methods. These approximations have definitely achieved speed gains by accommodating low-quality alignments in varying degrees. However, the speed limits of these algorithms will be challenged more seriously as the sequence capacity grows and will further test the balance between speed and accuracy of these processes.

This seemingly complex process of data analysis is dictated by the “simplistic” ideology of minimising the time and cost of data generation and interpretation. Driven by this ideology, it is imperative for researchers to adopt the fastest and the most accurate yet sensitive combination of prediction methods to analyse high throughput sequence data for variant discovery. Most large genome centres, like the ones involved in the 1000 genomes project [2], currently employ hundreds of informatics researchers to mine, analyse and interpret large NGS data sets. Analysis tools and algorithms developed by researchers working at these large centres may not always cater to the needs of an individual biologist who operates at a much lower capacity, scale and magnitude. Hence, there is a need to assess the existing freely available tools and algorithms for variant discovery and suggest a best combination to minimise time, informatics resources and subsequently reduce the number of false positives in the validation experiment. This will allow biologists focus more on their work rather than on optimising analytical tools for variant discovery from NGS data sets. Here, we present a comparative study of different freely available tools for short-read alignment and variant discovery that will allow individual biologists make a rational choice in plugging the fastest and accurate tool(s) into their data analysis pipeline.

The freely available short-read aligners sampled in our study belong to two major fundamental classes of algorithm implementation [3]. The first major class of aligners are built on hash table–based approach like; Bfast [4], Ssaha [5], Smalt [6], Stampy [7] and Novoalign [8] in which the hash is generated with the reference genome. The second class of aligners sampled are Burrows Wheeler transform (BWT)-based aligners Bwa [9] and Bowtie [10], which rely on creating an efficient index of the reference genome. Although the alignment algorithm plays a crucial role in variant calling, there are certain SNP callers that implement base quality and posterior probability calculations to minimize the false positive rates in the pool of variants called. We have mainly targeted the following standard and widely used SNP callers: Samtools [11], Freebayes [12], Bambino [13] and GATK [14], [15]. The methods section describes these tools in better detail in the context of our data.

**Figure 1:**
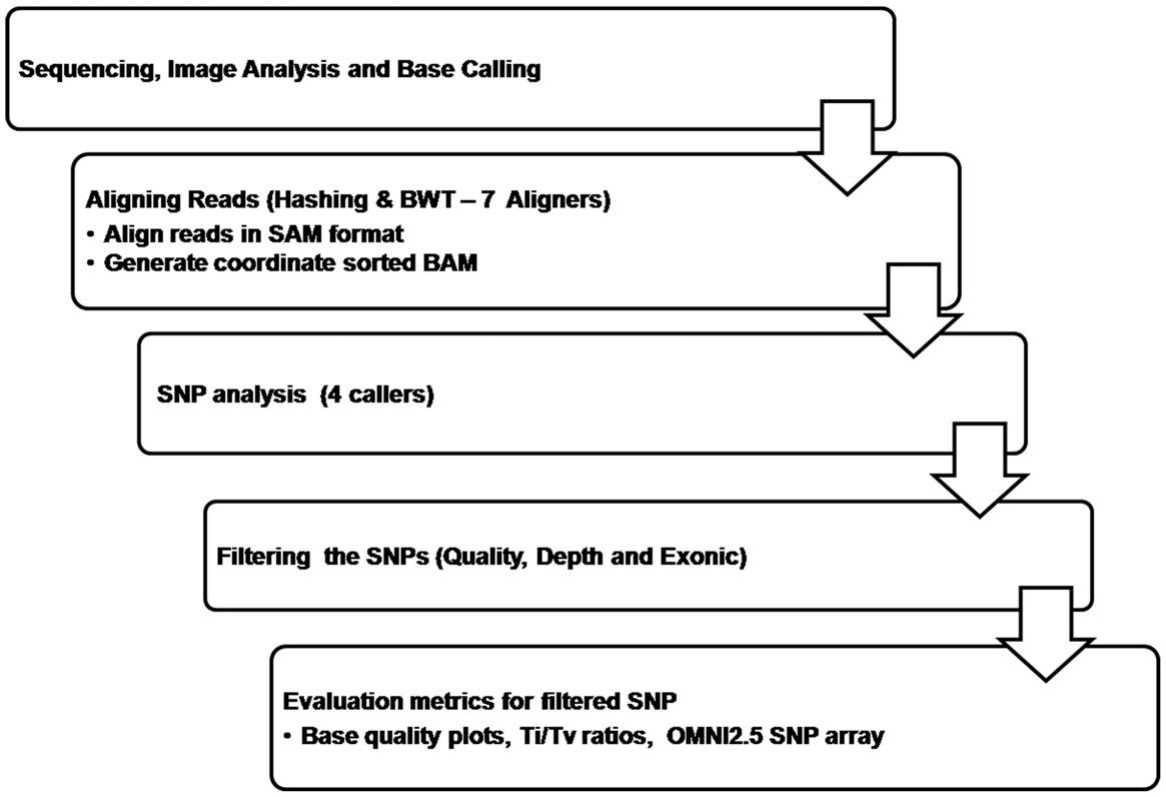
Steps involved in generating highly significant SNP dataset.

The steps involved in identification of a final set of SNPs from the whole exome data involves a number of steps (Figure 1) starting from the raw sequencing output. Each step shown in the schematic contributes to the accuracy of the final SNP calls. We decided to use real human whole exome data sets, derived from three different tissue samples, in both the alignment and SNP calling steps over simulated data to replicate biological variability and incorporate genome complexity to the analysis pipeline. The uncertainties in the resulting calls were treated in the downstream analysis using a user's perspective rather than emphasising on the complexity of the algorithms in practice. In our study, this was achieved by using a framework of simple pre-existing metrics like aligner and variant caller-specific base quality plots of the variants called, transition/transversion (Ti/Tv) ratios, SNP re-discovery rates using whole genome SNP microarray and run times associated with each tool. This study is aimed to facilitate biologists to choosing from the freely available resources for NGS exome data analysis.

## Results

Details on the sequencing platform used, short read aligners and variant callers assessed and the data sets used is given in Table 1. We have recorded useful insights centred around time associated with read alignment, quality of alignment and variant calling based on their quality scores and variant re-discovery rates from genotyping microarrays.

**Table 1:** The different NGS aligners and variant callers sampled in our study.

Sequencing platform	Aligners	SNP callers	Datasets
Illumina GAIIx, paired-end short-insert library of read length 76	Bowtie, Smalt, Stampy, Ssaha, Novoalign, Bwa, Bfast	Samtools,GATK, Freebayes, Bambino	Sureselect enriched Exome data: 02B, 12L, 20T

**Figure 2:**
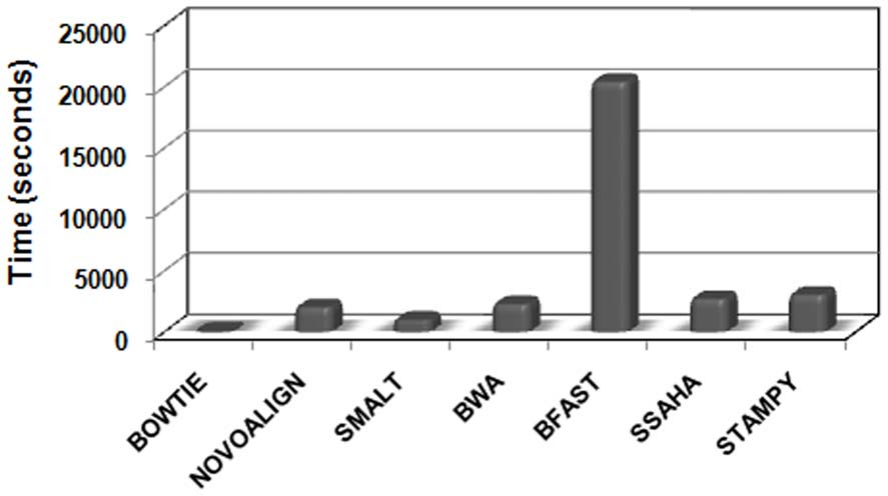
The real time elapsed in calculating alignment maps.

**Figure 3:**
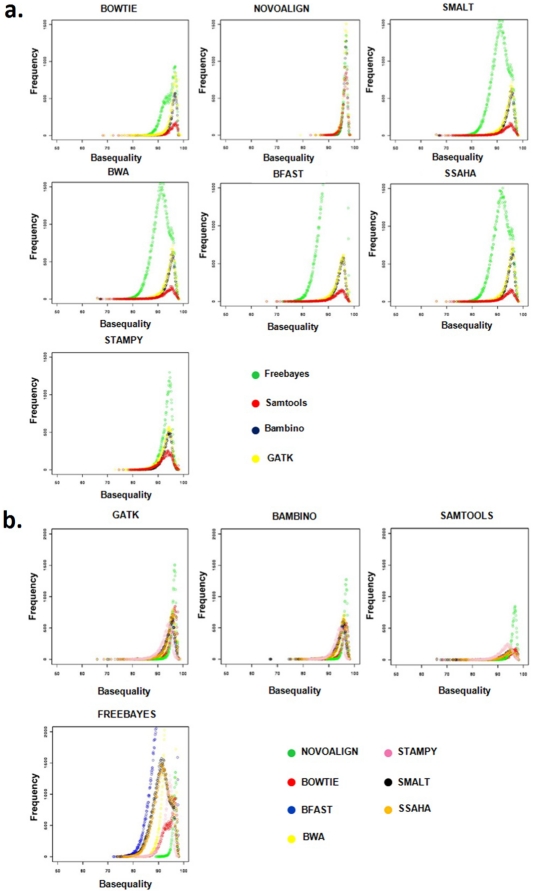
Base quality plots of sample 02B. (A) Depicting the effect of seven aligners. (B) Depicting the effect of four variant callers.

**Figure 4:**
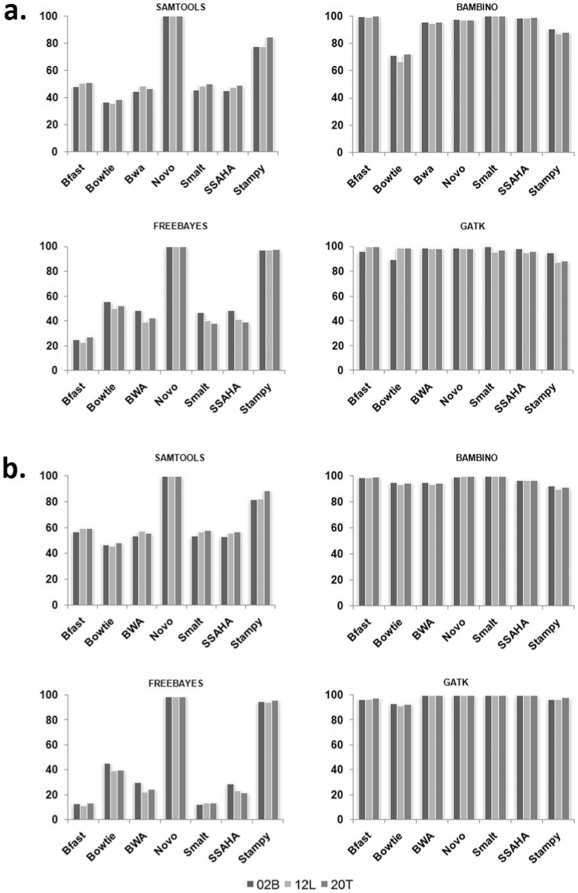
The variant rediscovery percentages determined using whole genome SNP array. (A) All exonic variants. (B) dbSNP positive variants. The Y axis represents the percent re-discovery rate in relation to the aligner that performed the best (taken as 100%).

Time taken for aligning reads by various aligners is represented in Figure 2. Bowtie, as expected, has the minimal run time as its alignment strategy does not accommodate gaps, essentially aligns only those reads bearing perfect matches and mismatches without insertions or deletions. Not surprisingly, the percentage of aligned reads in Bowtie is comparatively lower than any of the other aligners permitting gaps in calculating alignment maps (see Mapping Statistics in Text S1). Although not very sensitive in SNP detection, Bowtie's accuracy is very high as indicated by the metrics (Figures 3 and 4). However, earlier studies and preliminary studies from our group (data not shown) suggest higher SNP density around indel locations [16], it was not surprising to find that Bowtie captures only about 1% of SNP events in the vicinity of indels compared to Novoalign. In comparison to Bowtie, other aligners use gapped alignment approach but are efficient in time requirement. BFAST takes substantially more time in comparison to all other aligners and have a large RAM requirement [4], especially when 10 indexes are used for human reference genome. Although Stampy uses BWA algorithm in its first stage of alignment, the subsequent steps that introduce base-calibration makes it a relatively time consuming process in generating the SAM files. However, the ability of Stampy to combine two fundamentally different algorithms makes it a superior aligner compared to BWA. It implicates BWA's Burrows-Wheeler data structure as a first stage to map highly repetitive reads that include sequence variation followed by its own algorithm for further improvement of accuracy and sensitivity [7].

The next metrics used in our study is the average variant base qualities. We obtained the average base quality score of the variants directly from the corresponding SAM files thereby circumventing the possibility of any variant caller related arbitration in terms of assigning base quality scores. In the first analysis for sample 02B, we kept the aligner constant and varied the variant caller (Figure 3A) and in the second, we kept the variant caller constant and varied the aligner (Figure 3B). The top two aligners obtained using this metrics are Stampy and Novoalign (Figure 3). For the other two samples, the base quality plots were very similar (Figures S1, S2, S3, S4). The base quality plots indicate the ability of both Novoalign and Stampy to maintain a consistently high quality score across different variant callers. As anticipated, due to ungapped alignment and less number of total reads getting aligned to the reference genome, Bowtie yielded least number of variants with ^3^90 quality scores across different variant callers (Figure 3A). The most interesting results came when we used Novoalign that resulted in equally good variant quality no matter which downstream variant caller is used. This could be due to a post-alignment base quality re-calibration method that Novoalign uses [17]. Smalt, Bwa, Bfast and Ssaha yielded comparable base quality scores for all variant callers. Stampy, like Novoalign, also uses post-alignment base-recalibration and yielded good quality scores for all variant callers, albeit with varying frequency (Figure 3A).

The third metrics that we looked at is the Transition (Ti)/Transversion (Tv) ratio [15]. Ti/Tv ratio is generally used to evaluate the quality of SNP calls and is reported to be between 2–2.2 and 2.8–3.0 for SNPs anywhere in the genome and in the coding region respectively [15], [18], [19]. The data on Ti/Tv ratio depict the consistency of Novoalign and Stampy across all the variant callers (Table 2). GATK performed the best in terms of Ti/Tv ratio followed by Bambino across all variant callers. This perhaps is due to the fact that GATK is known to perform recalibration of base quality for variant calling [14], [15]. Bambino [13] assigns a Bayesian quality score [20] to each variant call that it calculates by converting the Phred-scaled scores of the aligned SAM file to probability of error value [21]. Freebayes fared poorly across all aligners except for Novoalign and Stampy. This could have been due to post-alignment base quality recalibration process that both Novoalign and Stampy employs.

**Table 2:** The Ti/Tv ratios of 28 different aligner-caller combinations for samples 02B, 12L and 20T.

Ti/Tv for Exonic SNPs		BWA	BFAST	BOWTIE	STAMPY	NovoMPI	SMALT	SSAHA
**02B**	**Samtools**	3.78	3.59	4.12	3.28	3.22	3.49	3.53
	**GATK**	2.73	2.73	2.86	2.77	2.77	2.79	2.75
	**Freebayes**	0.32	0.402	0.62	2.37	2.70	0.29	0.30
	**Bambino**	2.56	2.55	2.89	2.8	2.87	2.62	2.59
**12L**	**Samtools**	3.52	3.46	4.08	3.29	3.24	3.41	3.46
	**GATK**	2.69	2.72	2.90	2.76	2.75	2.78	2.74
	**Freebayes**	0.25	0.34	0.525	2.26	2.63	0.22	0.23
	**Bambino**	2.27	2.43	2.86	2.71	2.85	2.52	2.47
**20T**	**Samtools**	3.80	3.45	3.99	3.30	3.30	3.38	3.47
	**GATK**	2.74	2.73	2.85	2.75	2.77	2.78	2.75
	**Freebayes**	0.32	0.402	0.62	2.37	2.70	0.29	0.30
	**Bambino**	2.24	2.33	2.72	2.70	2.82	2.31	2.26

GATK produced the most high quality variants as depicted by the Ti/Tv ratios and the base quality plot metrics. Although, the number of steps involved in running GATK makes it a time intensive process, the steps for base call recalibration and local realignment [14] greatly improve the quality of call sets even in average quality alignment data. However, in our hands, Bambino proved to be faster yet accurate option for SNP calling with the quality of SNPs comparable to the GATK. Samtools resulted in a slightly higher (>3.0) Ti/Tv ratio suggesting a bias towards identifying transition events (Table 2). The pitfall of Samtools is that it uses very stringent quality filters and hence the probability of losing true positives in samtools is higher than the rest. The false detection fraction of variants in the call set has been deduced from the expected Ti/Tv ratio and an observed Ti/Tv from each call set (Table S1). A recent paper by Asan et al, suggests that in exome data the false positive rate is higher than false negative. Hence, we anticipate the discrepancy between the true positives and the variants discovered in our study could be primarily due to false positive rates [22].

The last metrics that we look into is the SNP re-discovery rate using whole-genome genotyping microarrays. We used whole genome SNP microarray from Illumina with 2.5 million SNPs (Omni 2.5 arrays). The number of variants re-discovered by SNP arrays and the overlap between the exome sequencing and SNP arrays is presented in Figure 4. SNP-rediscovery using microarray corroborated our earlier findings from previous metrics of average variant base quality and Ti/Tv ratio suggesting that both Novoalign and Stampy provide with the best rate of SNP re-discovery across all the variant callers (Figure 4A). The DNA microarray used in our study interrogates the unique regions of the genome. In order to validate the pipeline for novel variants, in addition to all the exonic SNPS, we calculated the variant re-discovery rates for the dbSNP positive variants from the whole genome SNP microarrays. As presented in Figure 4B, both Novoalign and Stampy provided the best rate of SNP re-discovery across all variant callers suggesting these aligners are equally useful to detect novel true positive variants.

## Discussion

In high-throughput sequencing, the most critical step, post sample/library preparation, involves accurate calculation of the alignment maps for reads with inexact matches to facilitate sensitive detection of biological variants by filtering out sequencing errors by a coverage based filter. Also, an inherent read mapping bias favoring the reference allele reduces the detection sensitivity of heterogeneous SNPs. The SNP masking approaches to limit the allele specific mapping bias are also not full proof [23]. Most of the aligners are challenged by the above limitation wherein the algorithms tend to lose true positives due to under mapping of reads with inexact matches and allele specific mapping bias. Read mapping biases resulted by aligning reads to a genome without masking the dbSNP variants is known to affect allele-specific expression [23] but its effect on variant calling remains to be established.

From a practical point of view, considering the cost, complexity of analysis, informatics load and the fact that the majority of disease-causing variants will remain within the coding region, it makes sense to utilize the whole-exome data sets over the whole genome ones. Although results presented here use whole exome data sets, we believe that the trend will hold good even for whole genome data sets.

From the data presented here, it is apparent that not the alignment *per se* but the post-alignment base quality recalibration plays an important role in true positive variant discovery. Both Novoalign and Stampy use this feature and hence yield a much better true-positive variant re-discovery rate, Ti/Tv ratio of variants called and higher base quality scores. In aligners where this feature is not enabled, we have got lower scores in all the three above metrics. Although, the post-alignment recalibration step has a predominant effect on the downstream analysis of aligned data, variant callers like GATK and Bambino have the ability to independently lower the false detection substantially. In our analyses, the deviation from the expected Ti/Tv ratio might be more of a reflection of false positive calls rather than false negative calls [15], [22]. The base quality plots (Figure 3B) are a graphic description of the prowess of the above tools in their ability to independently identify high quality variations.

In summary, we sampled 28 different combinations of aligners and variant callers in order to assess their ability to align sequence reads, obtain useful variant information, save time and cost of analysis and to come up with an optimised set of tools that can be used with minimum informatics support and resources. Among the tools tested, we funnelled down to four combinations involving two aligners Novoalign, Stampy, and two variant callers GATK and Bambino that provided the best variant quality and variant-rediscovery rates.

There are a wide variety of freely available tools for NGS short-read alignment and variant calling and we have sampled the most-common ones in order to identify the tangible combination under realistic computer hardware configurations and with reasonable time and cost required for follow-up validation. The metrics used in our study have independently and corroboratively suggest the accuracy and sensitivity of the above four combinations of tools at alignment and post alignment stage to reduce the number of false positive variants that can be taken for experimental validation in real whole exome data sets.

## Methods

### Generation of sequence data

Human samples were obtained after ethics committee approval from Mazumdar Shaw Cancer Centre, Narayana Hrudayalaya, Bangalore, India and after obtaining written informed consent from all participants involved in this study.

Illumina GAIIx was used to sequence three independent human samples. We decided to use GAIIx as it is by far the most popular NGS platform used in high-throughput sequencing studies. Although, most of the larger laboratories are migrating towards the newer HiSeq instruments, most small labs will remain with the GAIIx system for a foreseeable future as the amount of data from HiSeq is overwhelming to a biologist with limited hardware and informatics resources and support. Also, the quality of data generated from the GAIIx are comparable with that from the HiSeq systems, making our study relevant for data sets generated using other Illumina instruments using sequencing-by-synthesis chemistry. Additionally, if the error-associated with upstream sample/library preparation and the source of error remain same, then the conclusions also should hold good for other sequencing platforms.

Whole exome Sureselect enrichment kits (38MB) from Agilent Technology were used to enrich exonic regions from all the three samples. Sequencing libraries were prepared following standard Illumina library preparation protocol for paired-end 76 bp reads. Raw fastq files for both reads were generated and used for alignment process.

### Alignment

**Figure 5:**
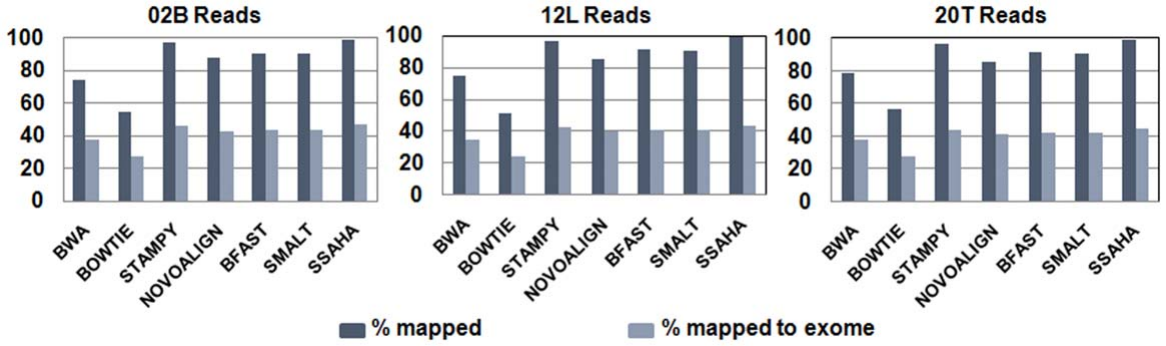
The alignment statistics of the percentage of reads aligned by different aligners.

To assess the time required to align equal number of raw sequence reads, we used a single core of the node in our HPC (IBM iDataPlex HPC with 48gb RAM) to perform alignment using all the aligners (this is to ensure that there is no difference between the tools tested that can multithread and the ones that can't, details on the command line arguments is given in Table S2). For all other analysis purpose, the alignment was carried out in a cluster environment exploiting the multi-threading feature (where available). In the case of those aligners wherein the multi/hyperthreading capacity was absent, we used in-house scripts to parallelise the input fastq file streams by splitting them into smaller chunks of size 0.5 GB and running them on individual cores to reduce time. This process of parallelization significantly improved speed of alignment. The smaller individual aligned SAM files spawned from the latter approach were merged to build the final aligned SAM file. All the aligners were instructed to generate aligned data in SAM format to facilitate downstream processing by multiple variant callers. The alignment statistics from different aligners are depicted by the graphs in Figure 5 (for details on the scripts and run parameters along with command line arguments used for each aligner, please see Alignment section under Text S1).

### SNP calling

Variant detection was done with Samtools, GATK, Freebayes and Bambino. Varying numbers of SNPs were detected by each caller (Tables S3, S4, and S5), which were filtered using exonic boundary limits to concentrate the SNPs in the exome region. As observed earlier in Figure 5, the percentage of reads aligned to the exome is lower than the percentage of total number of aligned reads indicating contamination/bleed over of reads generating from the non-coding region of the genome. The SNP data was generated using default parameters for each SNP caller. The workflow to call and filter variants involved in creating the final variant call set are reported in the supplementary section (see under SNP calling in Text S1). In-house Perl scripts were used to extract the corresponding average base qualities of the filtered SNPs and plotted using R script. The plots in Figure 3A and 3B depict the effect of aligner and variant caller in modulating the base quality of the variant call set.

### Transition/Transversion ratio (Ti/Tv)

A transition mutation involves a change from purine to purine or pyrimidine to pyrimidine and a tranversion mutation involves a change from pyrimidine to purine or *vice versa*. This makes a transversion event twice as favourable as a transition event for any random mutation event. Hence, the Ti/Tv ratio for a random variation resulting from systematic errors in the sequencing technology, alignment artefacts and data processing failures should be close to 0.5.

In our study, which involves targeted resequencing of the exonic regions of the genome, the expected range of Ti/Tv ratio is between 2.8–3.0 [19], [20]. The observed Ti/Tv ratios for each aligner-caller combination are tabulated/plotted in Table 2.

### Whole genome SNP microarray validation

Illumina OMNI2.5 whole genome SNP array was used to validate the SNP re-discovery rates from the whole exome sequencing experiments. The rediscovered SNPs are an experimental validation of the prowess of the NGS tools in contention. The use of SNP microarray allowed us to ascertain the percentage of true positives associated with each aligner and variant caller. The SNP sets compared here were filtered using exonic boundaries used for the sequencing experiment. The different percentages of overlap in all the 28 different combinations are shown in Figure 4.

### Note added to the proof

When this manuscript was under review, a report on comparative analysis of various mapping programs on read alignment was published [24]. Based on this, we extended our study using MAPQ filter cutoff of > = 30 and assessed its effect on the quality of the variant calls. The results obtained post MAPQ filtering did not change the overall results obtained earlier. Details of this analysis are provided in Table 6.

## Supporting Information

Figure S1 **Base quality plots of sample 12L depicting the effect of seven aligners.**
(TIF)

Figure S2 **Base quality plots of sample 12L depicting the effect of four variant callers.**
(TIF)

Figure S3 **Base quality plots of sample 20T depicting the effect of seven aligners.**
(TIF)

Figure S4 **Base quality plots of sample 20T depicting the effect of four variant callers.**
(TIF)

Table S1 **False Detection Rate table from the Ti/Tv metric.**
(PDF)

Table S2 **The elapsed real time output estimated by time command for each aligner.**
(PDF)

Table S3 **Number of raw SNP calls, filtered SNP calls (based on variant quality and depth) and the constituent exonic SNPs after applying Agilent SureSelect boundary filter for sample 02B.**
(PDF)

Table S4 **Number of raw SNP calls, filtered SNP calls (based on variant quality and depth) and the constituent exonic SNPs after applying Agilent SureSelect boundary filter for sample 12L.**
(PDF)

Table S5 **Number of raw SNP calls, filtered SNP calls (based on variant quality and depth) and the constituent exonic SNPs after applying Agilent SureSelect boundary filter for sample 20T.**
(PDF)

Table S6 **Metrics after applying the filter of MAPQ filter  = >30.** (A) With samtools. (B) With GATK. (C) With Freebayes. (D) With Bambino.
(PDF)

Text S1 **Parameters used for different aligners and for downstream analysis.**
(PDF)
