# Supplementary figures and images for: Customisation of the Exome Data Analysis Pipeline Using a Combinatorial Approach
Source: PLoS One. 2012 Jan 6;7(1):e30080. doi: 10.1371/journal.pone.0030080 (PMC3253117; doi:10.1371/journal.pone.0030080)

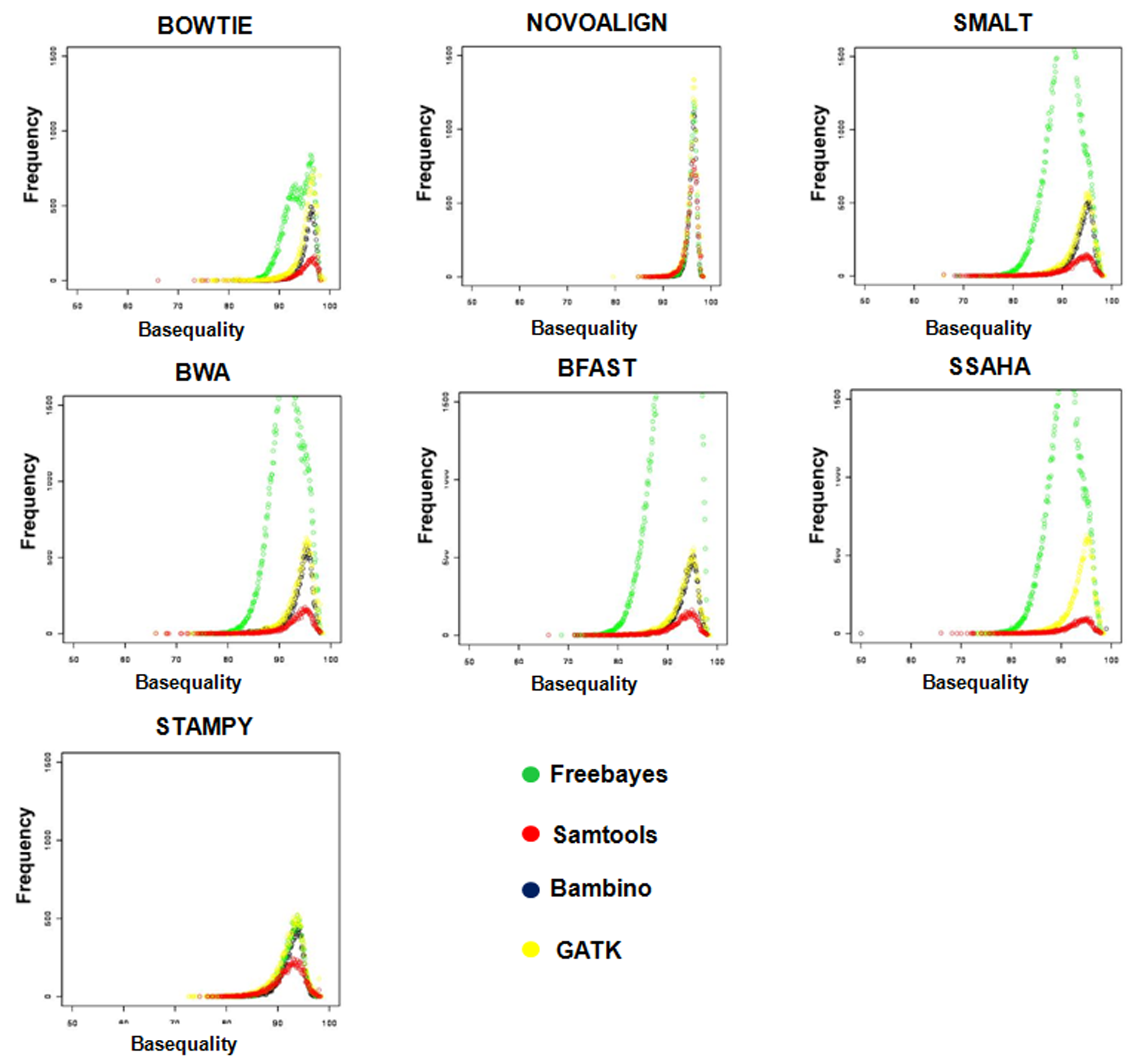

Supplement: Figure S1 — Base quality plots of sample 12L depicting the effect of seven aligners. (TIF) [file pone.0030080.s001.tif]

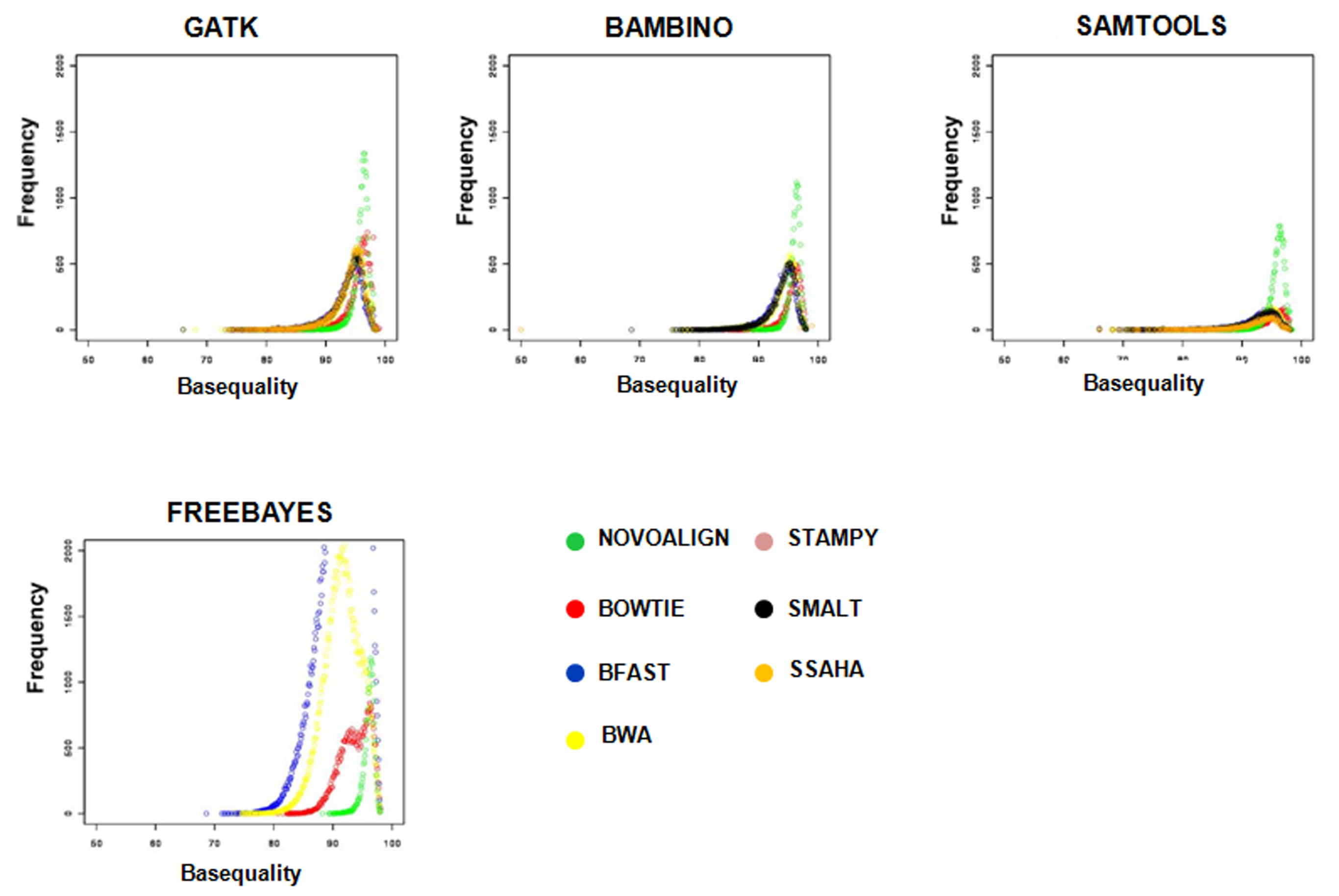

Supplement: Figure S2 — Base quality plots of sample 12L depicting the effect of four variant callers. (TIF) [file pone.0030080.s002.tif]

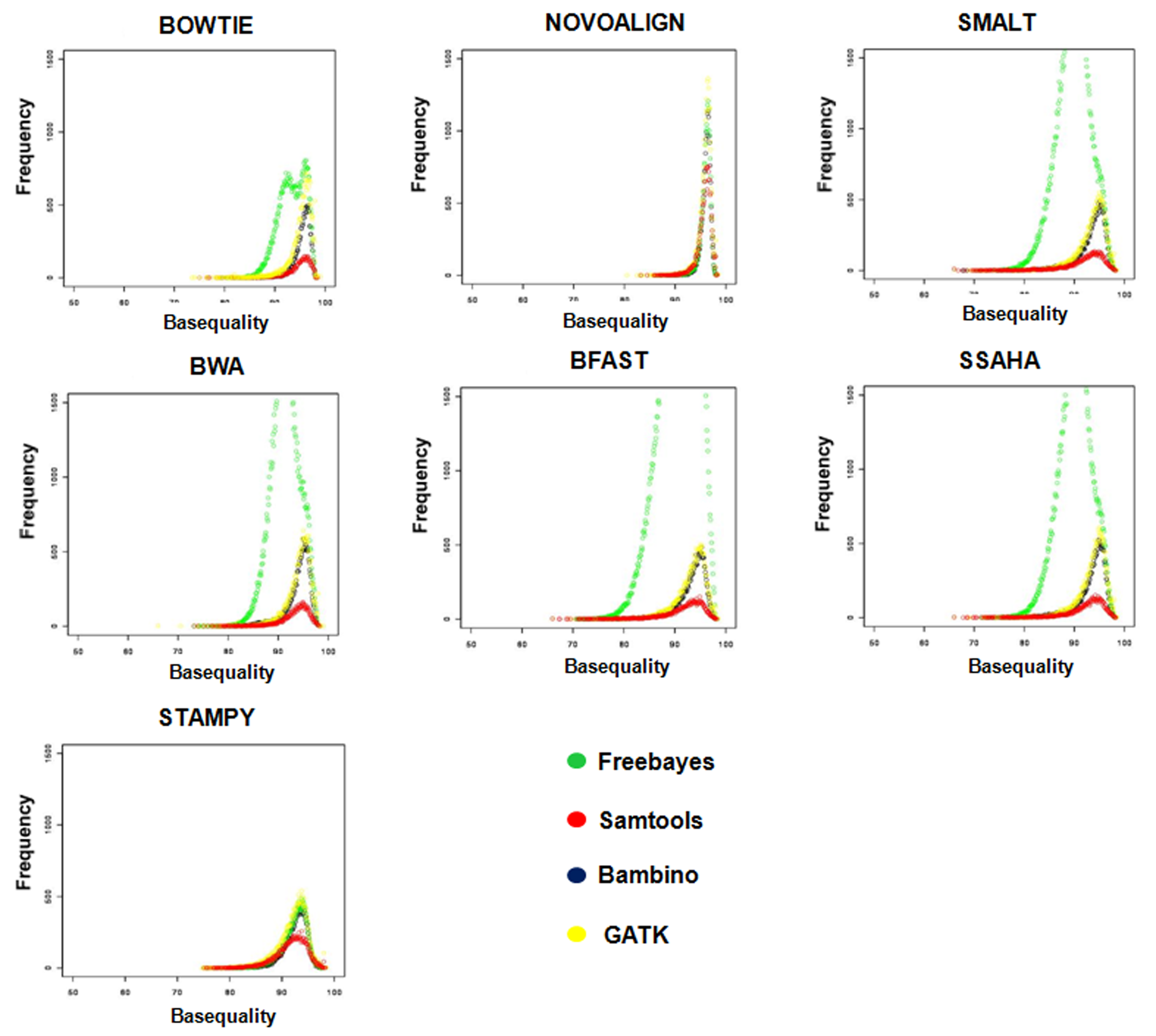

Supplement: Figure S3 — Base quality plots of sample 20T depicting the effect of seven aligners. (TIF) [file pone.0030080.s003.tif]

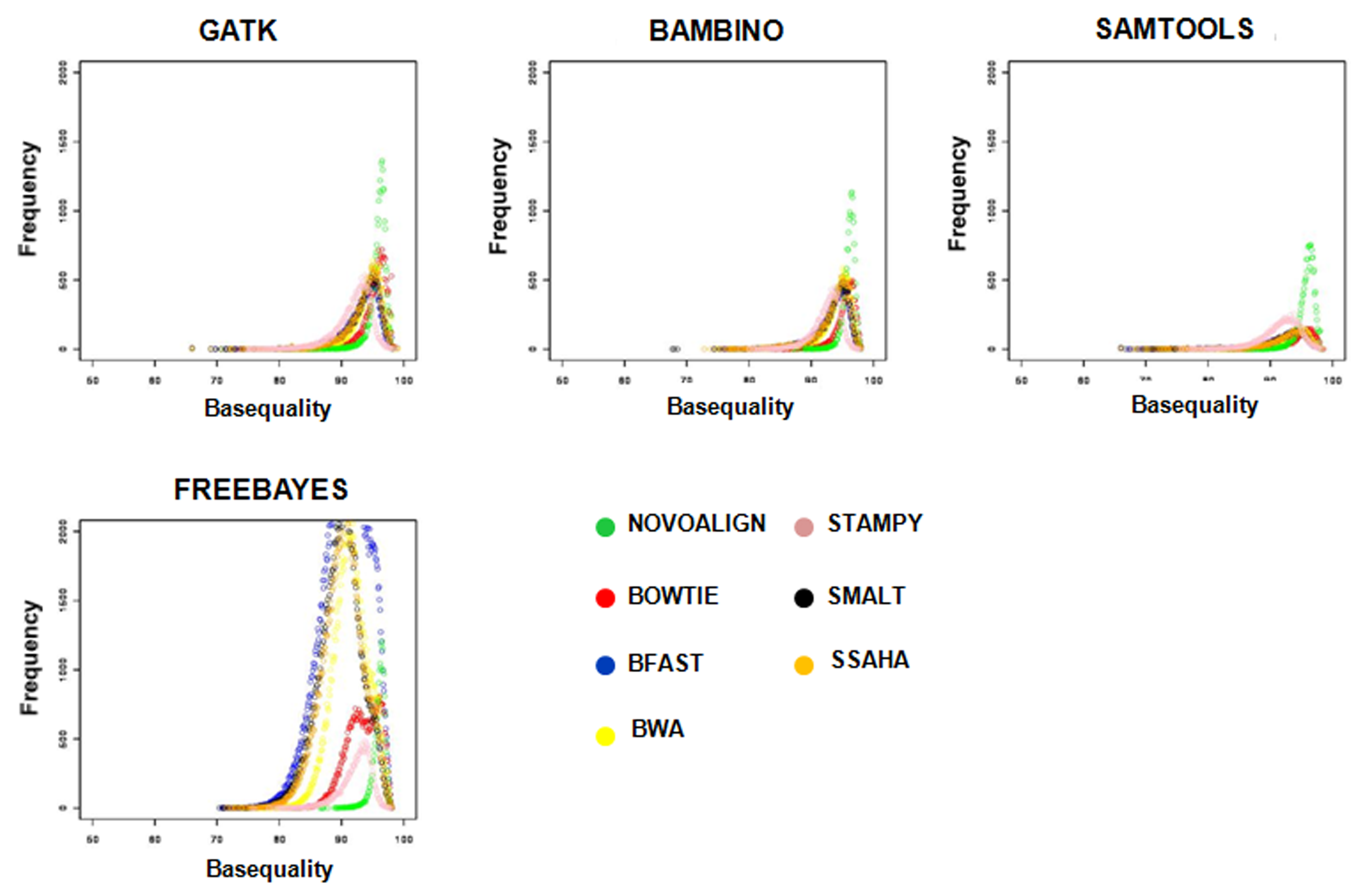

Supplement: Figure S4 — Base quality plots of sample 20T depicting the effect of four variant callers. (TIF) [file pone.0030080.s004.tif]
