# Supplementary material for: Customisation of the Exome Data Analysis Pipeline Using a Combinatorial Approach
Source: PLoS One. 2012 Jan 6;7(1):e30080. doi: 10.1371/journal.pone.0030080 (PMC3253117; doi:10.1371/journal.pone.0030080)
Supplement: Table S1 — False Detection Rate table from the Ti/Tv metric. (PDF) [file pone.0030080.s005.pdf]

Table S1: False Detection Rate table from the Ti/Tv metric.

**Aligners**	**Sample**	**GATK**	**Samtools**	**Freebayes**	**Bambino**
**BWA**	02B	0.89	1.31	0.00	0.83
	12L	0.88	1.21	0.00	0.71
	20T	0.90	1.32	0.00	0.70
**Bowtie**	02B	0.94	1.45	0.05	0.96
	12L	0.96	1.43	0.01	0.95
	20T	0.94	1.40	0.05	0.89
**Bfast**	02B	0.89	1.24	0.00	0.82
	12L	0.89	1.18	0.00	0.78
	20T	0.89	1.18	0.00	0.73
**Novo**	02B	0.91	1.09	0.88	0.95
	12L	0.90	1.10	0.85	0.94
	20T	0.91	1.12	0.88	0.93
**SSAHA**	02B	0.90	1.21	0.00	0.84
	12L	0.90	1.18	0.00	0.79
	20T	0.90	1.19	0.00	0.70
**Smalt**	02B	0.92	1.20	0.00	0.85
	12L	0.91	1.16	0.00	0.81
	20T	0.91	1.15	0.00	0.73
**Stampy**	02B	0.91	1.11	0.75	0.92
	12L	0.90	1.12	0.70	0.89
	20T	0.90	1.12	0.75	0.88
