# Supplementary material for: Customisation of the Exome Data Analysis Pipeline Using a Combinatorial Approach
Source: PLoS One. 2012 Jan 6;7(1):e30080. doi: 10.1371/journal.pone.0030080 (PMC3253117; doi:10.1371/journal.pone.0030080)
Supplement: Table S2 — The elapsed real time output estimated by time command for each aligner. (PDF) [file pone.0030080.s006.pdf]

Table S2: The elapsed real time output estimated by time command for each aligner.

Aligner	Command Line	Elapsed real (wall clock) time used by the process, in seconds.
Novocraft	novocraft/novoalign -d hg18_non_mpi.ndx -f 02B_1_6_1_split00_100Mb.fastq 02B_1_6_2_split00_100Mb.fastq -i PE 300,50 -a -F ILMFQ -o SAM	2019.67
Bowtie	bowtie-0.12.7/bowtie -q -n -a --best -p 1 -S hg18 -1 02B_1_6_1_split00_100Mb.fastq -2 02B_1_6_2_split00_100Mb.fastq	29.18
Bwa	bwa/bwa aln -t 1 hg_18.fa 02B_1_6_1_split00_100Mb.fastq	1067.58
	bwa/bwa aln -t 1 hg_18.fa 02B_1_6_2_split00_100Mb.fastq	1170.83
	/Apps/serial/bwa/bwa sampe hg_18.fa 02B_1_6_1_split00_100Mb.fastq.sai 02B_1_6_2_split00_100Mb.fastq.sai 02B_1_6_1_split00_100Mb.fastq 02B_1_6_2_split00_100Mb.fastq	25.24
Smalt	smalt/smalt_x86_64 map -i 250 -f sam -n 1 -o 02B_1_6_100mb_smalt.sam hg18k13s6_smalt 02B_1_6_1_split00_100Mb.fastq 02B_1_6_2_split00_100Mb.fastq	1002.66
Ssaha	SSaha/ssaha2 -solexa -skip 6 -pair 20,400 -outfile 02B_1_6_ssaha_100mb.sam -multi 1 -output sam -save hs36k13s6 02B_1_6_1_split00_100Mb.fastq 02B_1_6_2_split00_100Mb.fastq	2693.94
Stampy	Python2.6/bin/python stampy-1.0.12/stampy.py --bwa --bwaoptions=-q10 hg18.fa -g hg18 -h hg18 --gapopen=40 --gapextend=3 --bwamaxmismatch=-1 --readgroup=ID:02B,SM:exome,PL:illumina --solexa -f sam -o stampy_02B_split_100mb.sam -M 02B_1_6_1_split00_100Mb.fastq 02B_1_6_2_split00_100Mb.fastq	3025.63
Bfast	/Apps/parallel/bfast/bin/bfast match -f hg_18.fa -n 1 -K 8 -M 1280 -r 02B_1_6_1_split00_100Mb.fastq	2545.74
	/Apps/parallel/bfast/bin/bfast match -f hg_18.fa -n 1 -K 8 -M 1280 -r 02B_1_6_2_split00_100Mb.fastq	2583.42
	/Apps/serial/bfast+bwa/bin/bfast localalign -f hg_18.fa -n 1 -M 1280 -U -1 02B_1_6_1_split00_100Mb.fastq.bmf -2 02B_1_6_2_split00_100Mb.fastq.bmf	4263.84
	/Apps/serial/bfast+bwa/bin/bfast postprocess -f hg_18.fa -n 1 -i bfast.la_02B_1_6_split_12.baf -O 1	11011.78
