# Supplementary material for: Customisation of the Exome Data Analysis Pipeline Using a Combinatorial Approach
Source: PLoS One. 2012 Jan 6;7(1):e30080. doi: 10.1371/journal.pone.0030080 (PMC3253117; doi:10.1371/journal.pone.0030080)
Supplement: Table S3 — Number of raw SNP calls, filtered SNP calls (based on variant quality and depth) and the constituent exonic SNPs after applying Agilent SureSelect boundary filter for sample 02B. (PDF) [file pone.0030080.s007.pdf]

Tables S3: Number of raw SNP calls, filtered SNP calls (based on variant quality and depth) and the constituent exonic SNPs after sureselect boundary filter for sample 02B.

Aligner	Caller	# of SNPs Called	# of SNPs after Filtering	No of Exonic SNPs
BWA	Samtools	655274	46180	6808
	GATK	604335	-	25753
	Freebayes	16167543	162198	104234
	Bambino	168188	42987	20841
BFAST	Samtools	2263730	51059	7429
	GATK	726383	-	24266
	Freebayes	37500750	447855	286229
	Bambino	218126	39531	20742
BOWTIE	Samtools	186084	20988	5463
	GATK	290261	-	23074
	Freebayes	6076511	71147	43142
	Bambino	65796	23088	13825
STAMPY	Samtools	17255637	247568	12915
	GATK	756284	-	25068
	Freebayes	12439362	71111	22215
	Bambino	139330	37810	18977
NovoMPI	Samtools	1099115	266897	17430
	GATK	680079	-	26074
	Freebayes	4657723	56288	20539
	Bambino	175293	35685	19131
SMALT	Samtools	1457032	76906	7275
	GATK	651292	-	25335
	Freebayes	19439907	214381	121011
	Bambino	65796	23088	13825
SSAHA	Samtools	1240774	74222	7126
	GATK	670857	-	26453
	Freebayes	18543594	178714	114497
	Bambino	224508	46005	21644
