# Supplementary material for: Customisation of the Exome Data Analysis Pipeline Using a Combinatorial Approach
Source: PLoS One. 2012 Jan 6;7(1):e30080. doi: 10.1371/journal.pone.0030080 (PMC3253117; doi:10.1371/journal.pone.0030080)
Supplement: Table S4 — Number of raw SNP calls, filtered SNP calls (based on variant quality and depth) and the constituent exonic SNPs after applying Agilent SureSelect boundary filter for sample 12L. (PDF) [file pone.0030080.s008.pdf]

Table S4: Number of raw SNP calls, filtered SNP calls (based on variant quality and depth) and the constituent exonic SNPs after sureselect boundary filter for sample 12L.

Aligner	Caller	# of SNPs Called	# of SNPs after Filtering	No of Exonic SNPs
BWA	Samtools	1391002	60215	7746
	GATK	610524	-	25766
	Freebayes	22530966	224211	145248
	Bambino	199482	43389	20948
BFAST	Samtools	2776766	63946	8007
	GATK	765903	-	23952
	Freebayes	43915512	515969	331744
	Bambino	253707	39383	20258
BOWTIE	Samtools	482297	22729	5400
	GATK	290415	-	22069
	Freebayes	8369362	74479	46673
	Bambino	68012	20968	12514
STAMPY	Samtools	17405717	286170	13210
	GATK	813185	-	24822
	Freebayes	13493572	75967	21792
	Bambino	142409	35282	17812
NovoMPI	Samtools	1210524	307889	17739
	GATK	730492	-	25809
	Freebayes	5810605	59912	19961
	Bambino	198114	35044	18486
SMALT	Samtools	1967369	93105	7905
	GATK	700930	-	25049
	Freebayes	26189757	270530	151663
	Bambino	242723	39544	19935
SSAHA	Samtools	1780227	90171	7695
	GATK	728267	-	26299
	Freebayes	25022907	220899	142670
	Bambino	260772	46583	21235
