# Supplementary material for: Customisation of the Exome Data Analysis Pipeline Using a Combinatorial Approach
Source: PLoS One. 2012 Jan 6;7(1):e30080. doi: 10.1371/journal.pone.0030080 (PMC3253117; doi:10.1371/journal.pone.0030080)
Supplement: Table S5 — Number of raw SNP calls, filtered SNP calls (based on variant quality and depth) and the constituent exonic SNPs after applying Agilent SureSelect boundary filter for sample 20T. (PDF) [file pone.0030080.s009.pdf]

Table S5: Number of raw SNP calls, filtered SNP calls (based on variant quality and depth) and the constituent exonic SNPs after sureselect boundary filter for sample 20T.

Aligner	Caller	# of SNPs Called	# of SNPs after Filtering	No of Exonic SNPs
BWA	Samtools	818100	54159	7104
	GATK	638918	-	25417
	Freebayes	19720266	204875	131000
	Bambino	185875	46598	21336
BFAST	Samtools	2398445	61748	7981
	GATK	711615	-	24183
	Freebayes	39094993	411535	264101
	Bambino	218322	41237	20491
BOWTIE	Samtools	243165	23340	5669
	GATK	317185	-	22848
	Freebayes	7677818	84611	51466
	Bambino	69374	24358	13943
STAMPY	Samtools	13352476	266051	14116
	GATK	759198	-	25432
	Freebayes	11389360	72496	20956
	Bambino	126940	37178	18072
NovoMPI	Samtools	1179066	280981	17224
	GATK	688483	-	25711
	Freebayes	5111684	58383	19978
	Bambino	172007	36323	18565
SMALT	Samtools	1648858	85194	8080
	GATK	664296	-	25052
	Freebayes	24110143	285608	165389
	Bambino	214446	41855	20579
SSAHA	Samtools	1445024	82976	7832
	GATK	686781	-	26362
	Freebayes	23038163	243536	155998
	Bambino	231246	48785	21914
