# Supplementary material for: Customisation of the Exome Data Analysis Pipeline Using a Combinatorial Approach
Source: PLoS One. 2012 Jan 6;7(1):e30080. doi: 10.1371/journal.pone.0030080 (PMC3253117; doi:10.1371/journal.pone.0030080)
Supplement: Table S6 — Metrics after applying the filter of MAPQ filter = >30. (A) With samtools. (B) With GATK. (C) With Freebayes. (D) With Bambino. (PDF) [file pone.0030080.s010.pdf]

Table S6: Metrics after applying the filter of MAPQ filter =>30 with samtools (a), GATK (b), Freebayes (c) and Bambino (d).

a.

Aligner	# of SNPs called	# of SNP after Filtering	No. of Exonic SNPs	No. of SNPs after OMNI2.5 comparison	ti/Tv
Bwa	14653974	139807	93346	11422	0.33
Smalt	17737046	198668	114720	12153	0.31
Ssaha	16875126	166481	108859	12107	0.32
Novo	4702554	59759	20588	11474	2.70

b.

Aligner	# of SNPs called	# of SNPs after Filtering	# of Exonic SNPs	# of SNPs after OMNI2.5 comparison	Ti/Tv
BWA	616700	38464	6919	4087	3.82
Smalt	1352335	64585	7483	4255	3.54
Ssaha	1073396	62192	7587	4177	3.46
Novo	1123529	270370	17381	9625	3.23

c.

Aligner	# of SNPs called	# of SNPs after Filtering	# of Exonic SNPs	# of SNPs after OMNI comparison	Ti/Tv
BWA	122269	31832	18607	10944	2.62
Smalt	152793	31415	19031	11358	2.7
Ssaha	152751	34100	19713	11362	2.65
Novo	181075	33337	18682	11112	2.9

d.

Aligner	# of SNPs called	# of SNPs after Filtering	No. of SNPs after OMNI comparison	Ti/Tv
BWA	571120	24680	13832	2.835
Smalt	577925	24928	13914	2.784
Ssaha	623271	26163	13941	2.76
Novo	634390	25449	13906	2.8
