# Supplementary material for: Customisation of the Exome Data Analysis Pipeline Using a Combinatorial Approach
Source: PLoS One. 2012 Jan 6;7(1):e30080. doi: 10.1371/journal.pone.0030080 (PMC3253117; doi:10.1371/journal.pone.0030080)
Supplement: Text S1 — Parameters used for different aligners and for downstream analysis. (PDF) [file pone.0030080.s011.pdf]

**Text S1**

**Alignment:**

The aligners were run using the default run parameters as tabulated below:

BOWTIE (Multithreaded)– Version 0.12.7

```
bowtie-0.12.7/bowtie -q -n -a --best \
-p 12 \
-S hg18 \
-1 02B_****.fastq \
-2 02B_****.fastq
```

SMALT (Multithreaded) – Version 0.5.0.8

```
smalt/smalt_x86_64 map \
-i 250 \
-f sam \
-n 12 \
-o 02B_1_6_smalt.sam \
hg18k13s6_smalt 02B_****.fastq 02B_****.fastq
```

STAMPY – Version 1.0.12

```
Python2.6/bin/python stampy-1.0.12/stampy.py \
--bwa--bwaoptions=-q10 hg18.fa  --bwamaxmismatch=-1 \
-g hg18 -h hg18 \
--solexa --gapopen=40 --gapextend=3 \
--readgroup=ID:02B,SM:Exonic,PL:illumina \
-f sam \
-o 02B_1_6_stampy.sam \
-M 02B_****.fastq 02B_****.fastq
```

SSAHA – Version 2.5.3

```
SSaha/ssaha2 -solexa -skip 6 \
-pair 20,400 \
-outfile 02B_1_6_ssaha.sam -multi 1 -output sam \
-save hs36k13s6 02B_****.fastq 02B_****.fastq
```

NOVO (Multithreaded) – Version 2.07.00

```
mvapich2/bin/mpiexec.hydra \
-np 120 \
-hostfile /tmp/15174.1.all.q/machines novoalignMPI/novoalignMPI \
-d hg18.nidx \
-f 02B_****.fastq 02B_****.fastq \
-F ILMFQ \
-o SAM
```

BWA (Multithreaded) – Version 0.5.0

```
bwa/bwaaln -t 12 hg_18.fa 02B_****.fastq

bwa/bwaaln -t 12 hg_18.fa 02B_****.fastq

bwa/bwasampe hg_18.fa 02B_****.fastq.sai \
```

```
02B_****.fastq.sai 02B_****.fastq 02B_****.fastq
```

BFAST (Multithreaded) – Version 0.6.4e

```
bfast+bwa/bin/bfast match \
-f hg_18.fa \
-n 12 \
-K 8 \
-M 1280 \
-r 02B_****.fastq

bfast+bwa/bin/bfast match \
-f hg_18.fa \
-n 12 \
-K 8 \
-M 1280 \
-r 02B_****.fastq

bfast+bwa/bin/bfastlocalalign -f hg_18.fa \
-n 12 \
-M 1280 \
-U -1 02B_1_6_1.bmf -2 02B_1_6_2.bmf

bfast+bwa/bin/bfastpostprocess -f hg_18.fa \
-n 12 \
-i bfast.la_02B_1_6_12.baf -O 1
```

**Mapping statistics:**
Coordinate sorted BAM files were used to calculate the mapping statistics using samtools FLAGSTAT option.The mapping statistics for all the three samples are listed below:

02B_67_PE_BFAST+BWA
104513594 in total
0 QC failure
0 duplicates
94619529 mapped (90.53%)
104513594 paired in sequencing
52256797 read1
52256797 read2
65958150 properly paired (63.11%)
93859884 with itself and mate mapped
759645 singletons (0.73%)
1250636 with mate mapped to a different chr
1050920 with mate mapped to a different chr (mapQ>=5)

02B_67_PE_BOWTIE
104513594 in total
0 QC failure
0 duplicates
56620922 mapped (54.18%)
104513594 paired in sequencing
52256797 read1
52256797 read2

56620922 properly paired (54.18%)
56620922 with itself and mate mapped
0 singletons (0.00%)
0 with mate mapped to a different chr
0 with mate mapped to a different chr (mapQ>=5)

02B_67_PE_BWA
104513594 in total
0 QC failure
0 duplicates
77857651 mapped (74.50%)
104513594 paired in sequencing
52256797 read1
52256797 read2
68352756 properly paired (65.40%)
74896292 with itself and mate mapped
2961359 singletons (2.83%)
401394 with mate mapped to a different chr
205647 with mate mapped to a different chr (mapQ>=5)

02B_67_PE_SSAHA
104513594 in total
0 QC failure
0 duplicates
103421367 mapped (98.95%)
104513594 paired in sequencing
52256797 read1
52256797 read2
102133398 properly paired (97.72%)
102687842 with itself and mate mapped
733525 singletons (0.70%)
506628 with mate mapped to a different chr
469954 with mate mapped to a different chr (mapQ>=5)

02B_67_PE_STAMPY
104513594 in total
0 QC failure
0 duplicates
101148024 mapped (96.78%)
104513594 paired in sequencing
52256797 read1
52256797 read2
95959934 properly paired (91.82%)
98954198 with itself and mate mapped
2193826 singletons (2.10%)
743314 with mate mapped to a different chr
486922 with mate mapped to a different chr (mapQ>=5)

02B_67_PE_NOVO
104513594 in total
0 QC failure

0 duplicates
89324754 mapped (85.47%)
104513594 paired in sequencing
52256797 read1
52256797 read2
86699938 properly paired (82.96%)
87193136 with itself and mate mapped
2131618 singletons (2.04%)
291850 with mate mapped to a different chr
289297 with mate mapped to a different chr (mapQ>=5)

12L_12_PE_BFAST+BWA
105457878 in total
0 QC failure
0 duplicates
95883498 mapped (90.92%)
105457878 paired in sequencing
52728939 read1
52728939 read2
66500150 properly paired (63.06%)
95073252 with itself and mate mapped
810246 singletons (0.77%)
1271390 with mate mapped to a different chr
1039890 with mate mapped to a different chr (mapQ>=5)

12L_12_PE_BOWTIE
105457878 in total
0 QC failure
0 duplicates
53831898 mapped (51.05%)
105457878 paired in sequencing
52728939 read1
52728939 read2
53831898 properly paired (51.05%)
53831898 with itself and mate mapped
0 singletons (0.00%)
0 with mate mapped to a different chr
0 with mate mapped to a different chr (mapQ>=5)

12L_12_PE_BWA
105457878 in total
0 QC failure
0 duplicates
78993271 mapped (74.91%)
105457878 paired in sequencing
52728939 read1
52728939 read2
69601732 properly paired (66.00%)
76844672 with itself and mate mapped
2148599 singletons (2.04%)
403080 with mate mapped to a different chr

187411 with mate mapped to a different chr (mapQ>=5)
12L_12_pe_SSAHA
105457878 in total
0 QC failure
0 duplicates
104320834 mapped (98.92%)
105457878 paired in sequencing
52728939 read1
52728939 read2
102906154 properly paired (97.58%)
103463384 with itself and mate mapped
857450 singletons (0.81%)
508408 with mate mapped to a different chr
466105 with mate mapped to a different chr (mapQ>=5)

12L_12_PE_STAMPY
105457878 in total
0 QC failure
0 duplicates
101715760 mapped (96.45%)
105457878 paired in sequencing
52728939 read1
52728939 read2
96215096 properly paired (91.24%)
99122294 with itself and mate mapped
2593466 singletons (2.46%)
617554 with mate mapped to a different chr
393272 with mate mapped to a different chr (mapQ>=5)

12L_12_PE_NOVO
105457878 in total
0 QC failure
0 duplicates
89794993 mapped (85.15%)
105457878 paired in sequencing
52728939 read1
52728939 read2
86904500 properly paired (82.41%)
87397196 with itself and mate mapped
2397797 singletons (2.27%)
264042 with mate mapped to a different chr
260664 with mate mapped to a different chr (mapQ>=5)

20T_34_PE_BFAST+BWA
106526056 in total
0 QC failure
0 duplicates
96932990 mapped (90.99%)
106526056 paired in sequencing
53263028 read1
53263028 read2
73350624 properly paired (68.86%)

96136512 with itself and mate mapped
796478 singletons (0.75%)
1179996 with mate mapped to a different chr
987876 with mate mapped to a different chr (mapQ>=5)

20T_34_PE_BOWTIE
106526056 in total
0 QC failure
0 duplicates
60576324 mapped (56.87%)
106526056 paired in sequencing
53263028 read1
53263028 read2
60576324 properly paired (56.87%)
60576324 with itself and mate mapped
0 singletons (0.00%)
0 with mate mapped to a different chr
0 with mate mapped to a different chr (mapQ>=5)

20T_34_PE_BWA
106526056 in total
0 QC failure
0 duplicates
84051045 mapped (78.90%)
106526056 paired in sequencing
53263028 read1
53263028 read2
75183624 properly paired (70.58%)
80789554 with itself and mate mapped
3261491 singletons (3.06%)
392662 with mate mapped to a different chr
214917 with mate mapped to a different chr (mapQ>=5)

20T_34_PE_SSAHA
106526056 in total
0 QC failure
0 duplicates
105442298 mapped (98.98%)
106526056 paired in sequencing
53263028 read1
53263028 read2
104049282 properly paired (97.67%)
104617810 with itself and mate mapped
824488 singletons (0.77%)
520940 with mate mapped to a different chr
480385 with mate mapped to a different chr (mapQ>=5)

20T_34_PE_STAMPY
106526056 in total
0 QC failure
0 duplicates
102664452 mapped (96.37%)

106526056 paired in sequencing
53263028 read1
53263028 read2
96891594 properly paired (90.96%)
99947582 with itself and mate mapped
2716870 singletons (2.55%)
677432 with mate mapped to a different chr
418276 with mate mapped to a different chr (mapQ>=5)

20T_34_PE_NOVO
106526056 in total
0 QC failure
0 duplicates
91085059 mapped (85.50%)
106526056 paired in sequencing
53263028 read1
53263028 read2
88151256 properly paired (82.75%)
88634602 with itself and mate mapped
2450457 singletons (2.30%)
285728 with mate mapped to a different chr
282949 with mate mapped to a different chr (mapQ>=5)

**SNP calling:**
The command line options used in detecting SNPs using GATK, Samtools, Bambino and Freebayes are pasted below. The raw SNPs thus obtained were subject to filtration based on SNP quality and depth using in-house perl scripts. The final set of SNPs was obtained by extracting only those SNPs in the exonic regions. The sure select exon coordinates were used for the same.

Steps involved in calling SNPs using GATK version 1.0.5083 with default options:

1. java -Xmx8g –jar GATK/GenomeAnalysisTK-1.0.5083/GenomeAnalysisTK.jar \
   -R Homo_sapiens_assembly18.fasta \
   --default_platformillumina \
   --default_read_group '@RG\tID:s67\tSM:exonic\tPL:illumina' \
   --DBSNP dbsnp_129_hg18.rod \
   -I s_67_novompi_run9_coordSorted_merged.bam \
   -T CountCovariates \
   -covReadGroupCovariate \
   -covQualityScoreCovariate \
   -covCycleCovariate \
   -covDinucCovariate \
   -recalFile s_67_novompi_run9_coord_sorted_merged_GATK_op.first_run.recal.csv

2. java -Xmx8g –jarGATK/GenomeAnalysisTK-1.0.5083/GenomeAnalysisTK.jar \
   -R Homo_sapiens_assembly18.fasta \
   --default_platformillumina \
   --default_read_group '@RG\tID:s67\tSM:exonic\tPL:illumina' \
   -I s_67_novompi_run9_coordSorted_merged.bam \
   -T TableRecalibration \
   --out s_67_novompi_run9_coord_sorted_merged_GATK_op.first_recalibrated.bam \

-recalFile s_67_novompi_run9_coord_sorted_merged_GATK_op.first_run.recal.csv

3. samtools/samtools sort \
s_67novompi_run9_coord_sorted_merged_GATK_op.first_recalibrated.bam \
s_67_novompi_run9_coord_sorted_merged_GATK_op.first_recalibrated_sorted

4. samtools/samtools index \

s_67_novompi_run9_coord_sorted_merged_GATK_op.first_recalibrated_sorted.bam

5. java -Xmx8g -jar GATK/GenomeAnalysisTK-1.0.5083/GenomeAnalysisTK.jar \
-R Homo_sapiens_assembly18.fasta \
--default_platformillumina \
--default_read_group '@RG\tID:s67\tSM:exonic\tPL:illumina' \
--DBSNP dbsnp_129_hg18.rod \
-I
s_67_novompi_run9_coord_sorted_merged_GATK_op.first_recalibrated_sorted.bam \
-T CountCovariates \
-covReadGroupCovariate \
-covQualityScoreCovariate \
-covCycleCovariate \
-covDinucCovariate \
-recalFile
s_67_novompi_run9_coord_sorted_merged_GATK_op.second_run.recal.csv

6. java -Xmx8g -jar GATK/GenomeAnalysisTK-1.0.5083/GenomeAnalysisTK.jar \
-R Homo_sapiens_assembly18.fasta \
--default_platformillumina \
--default_read_group '@RG\tID:s67\tSM:exonic\tPL:illumina' \
-I
s_67_novompi_run9_coord_sorted_merged_GATK_op.first_recalibrated_sorted.bam \
-T TableRecalibration \
--out
s_67_novompi_run9_coord_sorted_merged_GATK_op.second_recalibrated.bam \
-recalFile
s_67_novompi_run9_coord_sorted_merged_GATK_op.second_run.recal.csv

7. samtools/samtools sort \
s_67_novompi_run9_coord_sorted_merged_GATK_op.second_recalibrated.bam \
s_67_novompi_run9_coord_sorted_merged_GATK_op.second_recalibrated_sorted

8. samtools/samtools index \
s_67_novompi_run9_coord_sorted_merged_GATK_op.second_recalibrated_sorted
.bam

9. java -Xmx8g -jar GATK/GenomeAnalysisTK-1.0.5083/GenomeAnalysisTK.jar \
-T RealignerTargetCreator \
-L \
-R Homo_sapiens_assembly18.fasta \
-D dbsnp_129_hg18.rod \
-o s_67_novompi_run9_coord_sorted_merged_GATK_op.intervals \
-I

s_67_novompi_run9_coord_sorted_merged_GATK_op.second_recalibrated_sorted.bam

10. java -Xmx8g -jar GATK/GenomeAnalysisTK-1.0.5083/GenomeAnalysisTK.jar \
    -I
s_67_novompi_run9_coord_sorted_merged_GATK_op.second_recalibrated_sorted.bam \
    -R Homo_sapiens_assembly18.fasta \
    -T IndelRealigner \
    -targetIntervals s_67_novompi_run9_coord_sorted_merged_GATK_op.intervals \
    -o s_67_novompi_run9_coord_sorted_merged_GATK_op.localy_realigned.bam

11. samtools/samtools sort \
    s_67_novompi_run9_coord_sorted_merged_GATK_op.localy_realigned.bam \
    s_67_novompi_run9_coord_sorted_merged_GATK_op.localyrealignedSorted

12. samtools/samtools index\
    s_67_novompi_run9_coord_sorted_merged_GATK_op.localyrealignedSorted.bam

13. java -Xmx8g -jar picard-tools-1.39/FixMateInformation.jar \
    INPUT=s_67_novompi_run9_coord_sorted_merged_GATK_op.localyrealignedSort
ed.bam \
    OUTPUT=s_67_novompi_run9_coord_sorted_merged_GATK_op.matepairfixed.ba
m \
    SO=coordinate \
    VALIDATION_STRINGENCY=SILENT

14. samtools/samtools sort \
    s_67_novompi_run9_coord_sorted_merged_GATK_op.matepairfixed.bam\
    s_67_novompi_run9_coord_sorted_merged_GATK_op.matepairfixed_sorted

15. samtools/samtools index \
    s_67_novompi_run9_coord_sorted_merged_GATK_op.matepairfixed_sorted.bam

16. java -Xmx8g -jar GATK/GenomeAnalysisTK-1.0.5083/GenomeAnalysisTK.jar \
    -R Homo_sapiens_assembly18.fasta \
    -T UnifiedGenotyper \
    -I s_67_novompi_run9_coord_sorted_merged_GATK_op.matepairfixed_sorted.bam
\
    -D dbsnp_129_hg18.rod -o
s_67_novompi_run9_coord_sorted_merged_GATK_op.raw.vcf \
    -stand_call_conf 50.0 \
    -stand_emit_conf 10.0 \
    -dco \
    -v 1000

17. java -Xmx8g -jar GATK/GenomeAnalysisTK-1.0.5083/GenomeAnalysisTK.jar \
    -T VariantAnnotator \
    -l INFO \
    -R Homo_sapiens_assembly18.fasta \
    -I s_67_novompi_run9_coord_sorted_merged_GATK_op.matepairfixed_sorted.bam
\
    -o s_67_novompi_run9_coord_sorted_merged_GATK_op.annotation.raw.vcf \
    -AAlleleBalance \

-A DepthOfCoverage \
-B:variant,VCF s_67_novompi_run9_coord_sorted_merged_GATK_op.raw.vcf

Steps involved in calling SNPs using SAMTOOLS version 1.12a with default options:

1. samtools/samtools view -bThg18.fa \ s_67_novompi_run9.sam > \
   s_67_novompi_run9.bam

2. samtools/samtools sort s_67_novompi_run9.bam
   s_67_novompi_run9_CoordSorted

3. samtools/samtools indexs_67_novompi_run9_CoordSorted.bam

4. samtools/samtools pileup -vcf hg18.fa \
   s_67_novompi_run9_CoordSorted.bam > \
   s_67_novompi_run9_CoordSorted_raw_pileup.txt

The raw pileup file was filtered using varFilter option and SNP quality filter of >= 20 using the SNP quality column($6^{th}$ column) of the ten column pileup formatted file.

Steps involved in calling SNPs using BAMBINO 1.01 with default options:

1. samtools/samtools view -bT hg18.fa  s_67_novompi_run9.sam > \
   s_67_novompi_run9.bam

2. samtools/samtools sort s_67_novompi_run9.bam
   s_67_novompi_run9_CoordSorted

3. samtools/samtools index s_67_novompi_run9_CoordSorted.bam

4. java –cpbambino/bambino.jar Ace2.SAMStreamingSNPFinder \
   -limit 1000 \
   -bam s_67_novompi_run9_CoordSorted.bam\
   -fasta ~/hg18.fa \
   -no-dbsnp \
   -of  s_67_novompi_run9_CoordSorted.bambinoOutput.txt

Steps involved in calling SNPs using FREEBAYES version 0.6.5 with default options:

1. samtools/samtools view –bT hg18.fa \ s_67_novompi_run9.sam > \
   s_67_novompi_run9.bam

2. samtools/samtools sort s_67_novompi_run9.bam
   s_67_novompi_run9_CoordSorted

3. samtools/samtools index s_67_novompi_run9_CoordSorted.bam

4. freebayes/freebayes \
   -b s_67_novompi_run9_CoordSorted.bam \
   -v s_67_novompi_run9_CoordSorted_FreebayesVCF.txt \
   -f ~/hg18.fa

**Ti/Tv metrics:**
The detection of false positives allowed us to filter out calls arising from natural bias of Ti/Tv, systematic errors, alignment artifacts and other data processing failures factored in the event of variant calling. This probability of a random call to be a false positive is assigned to be 0.5. The false detection rates were calculated using the formula below: FDRtest=(TiTvobserved − 0.5)/(TiTvexpected − 0.5).
We calculated the false-positive rates of all the 28 different aligner-caller combination from the Ti/Tv(observed) and Ti/Tv(expected) using the above formula .This allowed us to choose the combination of aligner-caller with lowest FDR (Table S1).
The false detection rates associated with the Ti/Tv ratios obtained by using the fraction stated in the methods section. The false detection rates obtained as a result of systematic errors, approximation in algorithm is expected to be closer to 0.1 (1).

**Average base quality plots:**
The trend in average base quality plots is maintained across the three different sampled used in our study. The base quality plots of samples 12L and 20T both with respect to aligners and callers are shown in Fig. S1-S4.

Detailed workflow to obtain the average base quality plot :

1. Run samtools pileup on the SAM files resulting from alignment :
   samtools pileup -f hg18.fa aligned.BAM > aligned.pileup_output
2. Filter the exonic SNPs from the variant caller(s) in contention
3. Use the average base quality of the filtered SNPs for each of the aligned SAM files: The average base quality was calculated from the sixth column of the aligned.pileup_output file
